# Exploring Urban Green Space Optimization of the Urban Walking Life Circle in Fuzhou, China

**DOI:** 10.3390/ijerph20021180

**Published:** 2023-01-09

**Authors:** Huili Xie, Xinke Wang, Xiaoting Hu, Zhiyong Shi, Hong Lin, Xiangqun Xie, Lingxiu Chen, Hongxia Dai, Jiao Zhang, Mengjie Xu, Xingzhao Liu

**Affiliations:** College of Landscape Architecture and Art, Fujian Agriculture and Forestry University, Fuzhou 350002, China

**Keywords:** urban green space, life circle, different research scales, equity, G2SFCA

## Abstract

The spatial distribution of urban green spaces (UGS) is closely related to the health of residents and the ecological pattern of cities. Exploring the equity of UGSs plays an important role in urban planning and also provides guidance for urban development. Taking the main urban area of Fuzhou City as an example, this study uses network big data and census data to pinpoint the population demand, evaluates the accessibility and equity of UGS within the basic living circle, neighborhood living circle and daily living circle of residents at the scale of residential and sub-districts. Based on the G2SFCA model, we also quantify the actual effective UGS’s service capacity. Then, using the scale and travel range as the entry point, we further discuss the similarities and differences under different scales and different travel ranges. Finally, optimization strategies are proposed for the construction status. The results show that: (1) The spatial allocation of urban green space resources varies significantly, and there is a serious inequity in the spatial distribution of urban green space under pedestrian conditions; (2) The results of UGS accessibility, equity, and service capacity in Fuzhou at both residential and sub-district scales are consistent; (3) Urban construction should be multi-level overall planning, combined with local economic and social development factors in accordance with local conditions to take measures. The results of the study can provide a scientific reference for the optimization of the spatial distribution of UGS.

## 1. Introduction

Urban green space (UGS) is an important part of urban natural ecosystems, which has the functions of purifying the environment [1], regulating microclimate [2], alleviating the urban heat island effect [3], and maintaining biodiversity and other ecosystem services [4]. It has many positive effects on human physiological and psychological health [5]. The justice of UGS resource allocation is not only conducive to enhancing the human well-being, but also promotes social justice and harmony [6]. Therefore, exploring the equity of UGS has an important role to play in urban planning and also provides guidance for urban development.

Most previous studies on UGS equity have started from accessibility, discussing whether residents have access to urban green space services within a certain spatial range. Accessibility was first proposed by Hansen in 1959 [7], and the existing measurement methods mainly include the buffer zone method, the minimum proximity method, the network analysis method, and the two-step floating catchment area method. Compared with other accessibility analysis methods, the two-step floating catchment area method takes into account the interaction between supply and demand, which was first proposed by R. John et al. [8] and it has been adopted by a large number of scholars since then. However, this method not only ignores the actual resistance of road network formation, but also does not consider the radius difference between the supply and demand side of the service [9,10]. To solve this problem, a series of improved algorithms have been derived from the two-step floating catchment area method. Shalini K et al. established the enhanced two-step floating catchment area model (E2SFCA) [11]; Polzin et al. proposed the expanded kernel density two-step floating catchment area (KD2SFCA) method and validated it in the Portuguese region [12]; and Weiji Xu [13], Xiaoyan Zhou et al. [14] used the gravity floating catchment area method (Gravity-2SFCA) to measure UGS accessibility. Xiaotong Xue et al. [15] used the Gaussian floating catchment area method (G2SFCA) to measure the urban accessibility of green space supply in different communities in Xuzhou. In previous studies, the demand of residents was mainly represented by population distribution data or census data, which is difficult to accurately match to the demand of the population. However, high-density cities lack space for development and construction. Accurate matching of supply and demand and urban micro renewal are new trends in urban planning.

Previous research scales mainly focused on macro-scales [16], but the evaluation of the mezzo-scale or micro-scale was insufficient, which led to many problems such as too-large units, inaccurate data, and so on. The existing studies lack comparative studies between different scales. In order to evaluate the equity of UGS more scientifically and accurately, this study takes the main area of Fuzhou City as an example, combines the data of the 7th Chinese census and network big data to locate the supply and demand, and adds a Gaussian decay coefficient on the traditional two-step floating catchment area (G2SFCA) method to measure the accessibility of UGS at different scales under various travel distances of residents in the main urban area of Fuzhou. At the same time, the Gini coefficient and Lorenz curve are introduced to evaluate the overall social equity of UGS allocation, and the effective coverage of UGS and the locational entropy method are used to discuss the real capacity of service supply of UGS. Previous studies lacked commonalities and diversities among different research scales and different travel scopes, so this study will also continue to improve and explore the spatial differentiation of UGS accessibility, equity and actual supply capacity of services at different research scales and different travel ranges. Then we will make planning proposals for urban construction in Fuzhou based on the results, with a view to providing a reference for the optimization of the spatial layout of UGS.

## 2. Materials and Methods

### 2.1. Study Area

**Figure 1:**
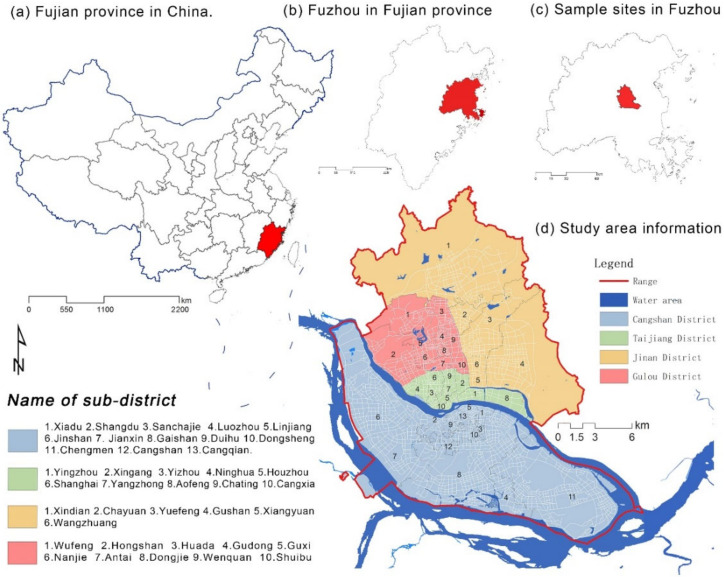
Location and information of study area.

Fuzhou, the capital of Fujian Province, is located at 25°15′~26°39′ N latitude and 118°08′~120°31′ E longitude, with a total area of 11,968 km^2^. It has a subtropical monsoon climate with suitable temperatures and warm and humid conditions. By the end of 2021, the total resident population in Fuzhou City was about 8.42 million, the regional GDP was 113.248 billion RMB, and the urbanization rate was 73.0% (access on http://tjj.fuzhou.gov.cn/zz/fztjnj/2022tjnj/zk/indexch.htm (accessed on 8 March 2022)). In this study, the main urban area of Fuzhou City was selected as the study area, with an area of about 311.8 km^2^ as of the end of 2021. It includes the whole area of Cangshan District, Gulou District and Taijiang District, and six sub-districts in Jin’an District, for a total of 39 sub-districts (Figure 1).

### 2.2. Data Source and Preprocessing

#### 2.2.1. Urban Green Space

**Figure 2:**
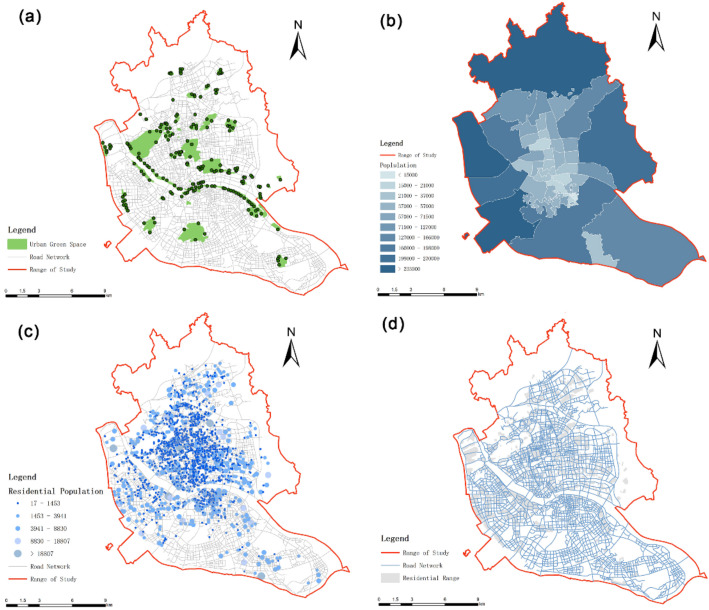
(**a**) Distribution of UGS in the study area. (**b**) Population distribution in sub-district scale. Information on the road network in the study area. (**c**) Population distribution in residential scale. (**d**) Information on the road network in the study area.

UGS is the green infrastructure of the city, as well as a basic spatial guarantee for sustainable urban development [17]. Previous studies mostly focus on park green space and mostly take the geometric center of green space as the service supply point, which ignores the service value of other types of UGS and is not in line with the actual situation. The Fuzhou Urban Green Space System Plan (2016–2020) divided UGS into five categories: park green space, square green space, protective green space, regional green space and attached green space. Since the protective green spaces focus more on ecological protection function and the attached green spaces were not developed externally leading to the specificity of the service population, only park green space, square green space and regional green space were included in this study. This study extracted 85 UGSs with a total area of about 76,537.6 ha with reference to the “Urban Green Line of Fuzhou City (2016–2020)”, published by the Fuzhou Landscape Bureau and Google remote sensing image data. We assumed that residents have access to the entrance of the UGS within a certain walking range that is served by the UGS. According to previous studies [13], the geometric centers of green areas with a scale of less than 5 ha were extracted and used as the center of the network analysis; for green areas with a scale larger than 5 ha, the actual entrances and exits of the green areas were determined by Baidu map poi data and field research (Table 1). The park green space dataset was established (Figure 2a).

#### 2.2.2. Residential Population Data

The population can represent the demand for urban green space to a certain extent. To obtain more accurate research results, this study used residential areas as the measurement unit for analysis. The outline of residential areas within the study area was obtained from Baidu Map (https://lbsyun.baidu.com/, accessed on 29 December 2021). The geometric center of each residential area was used as the service demand point, and a total of 2020 residential points were finally obtained. This study assumed that the population was evenly distributed within the settlement area [18]. The population of all settlements in each sub-district was calculated based on the population data of the sub-district (Figure 2b), and the proportion of each settlement in the total area of the settlement in its street (Figure 2c, Table 2). The population data of sub-districts were obtained from the data of the seventh national census published by the Fuzhou City Bureau of Statistics (http://tjj.fuzhou.gov.cn, accessed on 20 December 2021). The formula is as follows.

(1) $$D_{k}=\frac{RA_{K}}{RA}\times SP,$$

where *Dk* is the population in the kth residential area; *RAk* is the area of kth residential area; *RA* is the total residential area of the sub-district where the residential area is located; *SP* is the total population of that sub-district. Thirty residential areas were randomly selected as samples and, by surveying the number of households in these residential areas, the calculation results of the above formula were tested according to the average household size of 3.5 persons (the results of the seven popular population statistics in Fuzhou City). The average accuracy rate was at 76%, so the present estimation was considered to be able to estimate the residential population well.

#### 2.2.3. Road Network

Open Map Street platform was used to obtain open-source data (www.openstreetmap.org, accessed on 4 February 2022) and extract the road network data in and around the study area. The data were combined with Google Maps’ high-accuracy images to correct the data, screen out railways, highways and other roads that are not suitable for pedestrians. Finally, we obtained a road network that better matched the real situation (Figure 2d).

### 2.3. Methodology

**Figure 3:**
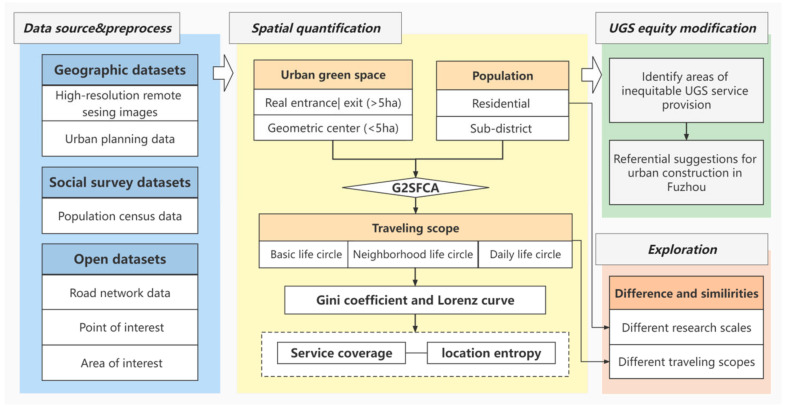
Technology Route.

Firstly, we used multi-source data to build a database of UGSs and population. After that, calculated the accessibility of residents under pedestrian condition based on G2SFCA model, and further explored the fairness of accessibility. We also quantified the service capacity of UGS using service coverage and locational entropy. Finally, we identify the inequitable areas of UGS service provision in Fuzhou and make reference suggestions for urban construction (Figure 3).

#### 2.3.1. G2SFCA-Based Accessibility Evaluation

The Gaussian two-step floating catchment area (G2SFCA) method is an accessibility research method optimized from the traditional two-step floating catchment area method. The distance decay coefficient is added to the traditional calculation, which is more suitable for the actual situation while the calculation results are more reliable. In this study, residents were set to reach UGS by walking, and the walking speed was determined to be 5 km/h according to previous studies [13]. The 5-min walking distance is the basic activity range of residents [19], and 15 min is the best travel time for residents to use the UGS service [20,21], while the ultimate travel time is 30 min [2,22]. So, this study delineated the 5-min living circle, 15-min living circle and 30-min living circle as the travel range (Table 3) to further quantify the residents’ received services by UGS under different activity ranges (Table 3).

The spatial accessibility evaluation based on G2SFCA was divided into two main steps.

In the first step, the UGS point data was used as the starting point and the residential points as the destination points for analysis, and the residential points *k* within the search space range *d*_0_ were filtered out from the UGS_j_. The number of all populations within d_0_ was aggregated and assigned weights according to the distance decay law using the Gaussian function [22]. These weighted populations were summed and aggregated to calculate the supply–demand ratio *Rj*.

(2) $$R_{j}=\frac{S_{j}}{\Sigma_{k\in \{d_{jk}⩽d_{0}\}}{}^{G\left(d_{jk}\right)D_{k}}}$$

where *D_k_* is the population number of each residential *k*, *d_kj_* is the actual road network distance between locations k and j. For the UGSs with multiple entrances, the road network distance from the settlement to the nearest entrance was selected, while the settlement *k* is in the search field (i.e., *d_kj_* ≤ *d*_0_); *S_j_* is the area of urban green space *j*; *G(d_kj_*) is a Gaussian decay function considering the spatial friction problem. Its expression as follows.

(3) $$G\left(d_{kj}\right)=\frac{e^{-\frac{1}{2}\times {\left(\frac{d_{kj}}{d0}\right)}^{2}}-e^{-\frac{1}{2}}}{1-e^{-\frac{1}{2}}}(d_{\mathrm{jk}}\text{<}d_{0}).$$

In the second step, the analysis was performed with residential points as the starting point and UGS point data as the destination point. All UGSs *j* in the search domain *d*_0_ were found separately, and the supply and demand ratios *R_j_* of these UGSs were summed up on the basis of Gaussian decay function to obtain *k* the spatial accessibility Ak based on the distance cost, whose larger value indicates a higher degree of accessibility. The formula is as follows:


(4) Ak=Σ
j∈dkj⩽d0GdkjRj

The accessibility results calculated by the G2SFCA have the meaning of UGS capita in a wide sense. Therefore, it can be compared with UGS per capita as a basis for evaluating accessibility, especially for horizontal comparison of different units in the region [23]. The UGS per capita is the ratio of urban green space to total population, and the measured green space per capita in the study area is 6.95 m^2^/person.

#### 2.3.2. Accessibility-Based Spatial Equity Evaluation

The Lorenz curve method is often used to explore the justice of national income in distribution. Recently, the Lorenz curve has been widely used in the fields of urban public resource allocation and spatial layout of green space to reflect the justice level. The value of Gini coefficient is taken between 0~1 [24]. The smaller the Gini coefficient, the more reasonable the level of spatial distribution of resources among urban residents and the fairer its spatial distribution. In this study, the Lorenz curve was introduced to analyze the justice level of the spatial distribution of UGS in the main urban area of Fuzhou. The calculation formula is as follows.

(5) $$G=1-\frac{1}{n}\left(2\Sigma_{i=1}^{n-1}W_{i}+1\right)$$

where *G* is the Gini coefficient, *n* is the total number of sub-districts (or residential areas) in the study area, ‘ *i*’ is the *i*-th sub-district (residential area) after the result of the ratio of the cumulative value of accessibility of each sub-district (residential area) to the number of people in the sub-district (residential area) is ranked from smallest to largest, *i* = 1, 2, …, *n*; *Wi* is the proportion of urban green space accessibility per capita from sub-district (residential area) 1 to sub-district (residential area) *i* to the total accessibility per capita in the whole area.

#### 2.3.3. Accessibility-Based Performance Evaluation of UGS Services

(1) UGS service coverage

Referring to the studies of Shuang Niu et al. [25] and Zilai Tang et al. [26], and we selected the ratio of the effective service area of UGS in a spatial unit (the sum of population) to the area of the spatial unit (population) as a quantitative indicator of the service level of UGS, i.e., the effective service area ratio and the effective service population ratio. The formulas are as follows:

(6) $$T_{j}=\frac{\sum S_{n}}{S_{j}}=\frac{S_{1}+S_{2}+S_{3}+\ldots +S_{n}}{S_{j}},$$

(7) $$U_{j}=\frac{\sum P_{n}}{P_{j}}=\frac{P_{1}+P_{2}+P_{3}+\ldots +P_{n}}{P_{j}},$$

where *T_j_* is the UGS effective service area ratio of unit *j*; *U_j_* is the UGS effective service population ratio of unit *j*; *n* represents the presence of *n* residential areas in sub-district *j*.

(2) Location entropy method

The locational entropy represents the ratio of UGS services received by the population living in a study unit to the per capita UGS resources of the residents in the whole study area. It can be used to express the spatial distribution pattern of the justice of the per capita distribution of resources in different study units. The higher the locational entropy value, the higher the level of resource allocation equipped in the study unit. If the locational entropy of the study unit is greater than 1, it indicates that the per capita level of UGS resources receiving services in the study unit is higher than the overall level of the whole study area; if the locational entropy is less than 1, it indicates that the per capita level of urban green space resources enjoyed in the study unit is lower than the overall level. The calculation formula is as follows:

(8) $$AALQ_{j}=\frac{Aj}{\left(\frac{S}{P}\right)},$$

where *A_j_* denotes the per capita UGS enjoyed by the population of unit j within the travel threshold, i.e., the actual accessibility of UGS obtained using the two-step floating catchment area method, *S* denotes the total UGS within the study area, *P* denotes the total population within the study area, and the ratio of *S* to *P* is the per capita UGS resources in the study area.

## 3. Results

### 3.1. Evaluation of Spatial Accessibility of UGS

#### 3.1.1. Residential Scale

Based on the G2SFCA method, the spatial accessibility of UGSs within the 5-min life circle, 15-min life circle and 30-min life circle of residential areas in the study area was analyzed. The results were graded by the natural breakpoint method into five grades: very low, lower, middle, higher and very high (Table 4).

A total of 1689 residential areas in the main urban area of Fuzhou are not accessible within the 5-min life circle, i.e., 83.6% of the residential areas in the study area cannot be served by UGSs; only 5.7% of the accessible residential areas are rated as higher and very high in accessibility, among which those with very high accessibility are located in the Xiangyuan sub-district and the Yingzhou sub-district. The accessibility of only 190 settlements is greater than the per capita UGS in the study area, accounting for 57.4% of the total accessible settlements. The mean value of the accessibility evaluation result is 40.2, while the standard deviation is 953.7, with obvious spatial variation (Table 5).

When the search radius was set to 15 min living circle, 70.1% of the settlements were inaccessible. The settlements with grade of inaccessible or very low in theHongshan sub-district, the Xindian sub-district, the Jianxin sub-district and the Gushan sub-district were the most numerous, with the number of settlements with both classifications exceeding 5% of the total number of settlements; settlements with very high accessibility were located in the Hongshan, Wenquan and Xindian sub-districts. There are 322 settlements with accessibility greater than the per capita green area in the study area, accounting for 53.4% of the total number of accessible settlements. The mean value of the settlement accessibility evaluation results is 35.8 in the 15-min life circle, and the standard deviation is 337.3, which also shows obvious spatial differentiation (Table 5).

When the search scope was expanded to the 30 min living circle, 55.1% of the residential spots in the study area were accessible; among them, 540 residential areas were accessible more than the UGS area per capita, accounting for 48.5%, which is a significant improvement in accessibility compared to the first two cases. However, there are still cases of inaccessible settlements. These are concentrated in the incompletely developed areas at the edge of the study area and in the relatively sparsely constructed areas of the Sanchajie and Duihu sub-districts.

**Figure 4:**
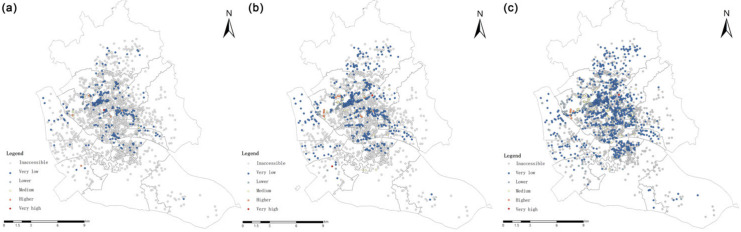
G2SFCA-based residential scale accessibility under different traveling scopes (**a**) 5-min life circle. (**b**) 15-min life circle. (**c**) 30-min life circle.

According to the results of UGS accessibility at the scale of residential area, the spatial separation of UGS occurs in the study area under the search radii of 500 m, 1000 m and 2000 m (Figure 4). The smaller the activity circle, the more green space service blind areas; even within the 30 min living circle, the percentage of residential areas within the service blind area reaches 44.9%. These indicate that the current UGS arrangement can hardly meet the population demand.

#### 3.1.2. Sub-District Scale

**Figure 5:**
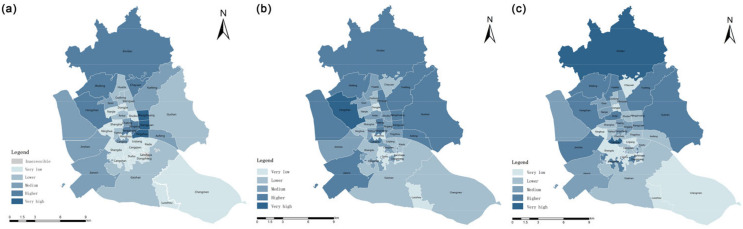
G2SFCA-based sub-district scale accessibility under different traveling scopes (**a**) 5-min life circle. (**b**) 15-min life circle. (**c**) 30-min life circle.

The accessibility analysis results of sub-district scale based on G2SFCA are shown in Figure 5: within the 5-min life circle, the Wangzhuang, Xiangyuan and Yingzhou sub-districts, which are located in the center of the study area, have the highest accessibility, while Sanchajie sub-district is beyond the accessibility space and has inaccessibility. In the case of the 15-min life circle, the number of sub-districts with high accessibility increases, accounting for one-third of the total. The accessibility of the Hongshan sub-district ranks first. Further expanding the search threshold to the 30 min living circle, the sub-districts with the highest accessibility are the Xindian sub-district and the Cangxia sub-district.

The overall accessibility of the study area showed as high in the northwest and low in the southeast under all three activity range circles. There are four sub-districts with unchanged grades, from higher, middle, lower to very low accessibility corresponding to Wufeng, Guxi, Gudong, and Luzhou sub-districts, respectively. The accessibility level of Shangdu sub-district and Chengmen sub-district has been at a very low or lower level. Eighteen percent of the sub-districts have changed significantly, with the increase of travel range, the accessibility levels of the Antai sub-district, Cangxia sub-district and Changshan sub-district have continued to become higher, while the accessibility levels of four sub-districts, namely Chayuan sub-district, Yingzhou sub-district, Wangzhuang sub-district and Xiangyuan sub-district, have been reduced.

### 3.2. Evaluation of Spatial Equity of UGS

**Figure 6:**
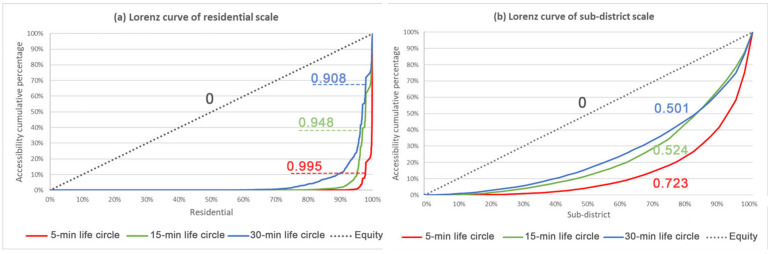
Gini coefficient and Lorenz curve under different scales. (**a**) Residential scale. (**b**) Sub-district scale.

The Gini coefficient and Lorenz curve express the overall social equity performance of UGS allocation (Figure 6). The calculated results of the Gini coefficient under the residential scale are all greater than 0.9. The UGS resource allocation varies greatly among residential areas and the social inequity is significant. The spatial allocation of UGS in the study area at the sub-district scale has a large gap in each sub-district, and all three spatial thresholds show a large difference in the allocation of UGS resources; especially at the search radius of the 15-min life circle, the Gini coefficient achieves 0.723, indicating the serious social inequity. Both research scales reveal significant differences in the allocation of UGS and serious social inequity.

### 3.3. Service Performance Evaluation of UGS

#### 3.3.1. Residential Scale

**Figure 7:**
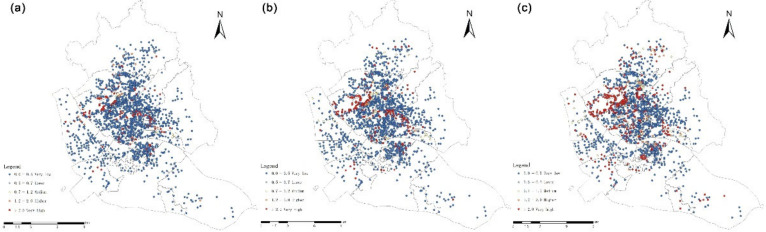
Locational entropy at residential scale under different traveling scopes (**a**) 5-min life circle. (**b**) 15-min life circle. (**c**) 30-min life circle.

The location entropy reflects the specific pattern of “spatial matching” between the allocation of UGS and the resident. Additionally, the sub-district location entropy value grade generally represents the evaluation level of the actual per capita UGS allocation. Therefore, after measuring the fairness in general, the spatial distribution pattern of UGS social equity can be analyzed using the locational entropy method (Figure 7). With reference to previous literature, the locational entropy value is divided into five grades: very low, lower, medium, higher, and very high. The locational entropy grading under the two research scales is counted separately [27].

Based on the locational entropy method, it was calculated that 9.5%, 16.0% and 26.8% of the settlements had a higher per capita acceptance level of UGS than the overall level of the study area at 500 m, 1000 m and 2000 m search radii, respectively. A hierarchical classification was made for each residential area. As can be seen from Table 6, the total number of settlements with very low and lower locational entropy tends to decrease as the spatial threshold increases, while the number of settlements with higher and very high locational entropy increases significantly. This indicates that, within a certain range, an increase in the actual choice of the travel distance of residents will result in more residents accepting the UGS. However, even at the travel limit distance (2000 m) about 70.6% of residential areas receive a lower or very low level of UGS services.

#### 3.3.2. Sub-District Scale

**Figure 8:**
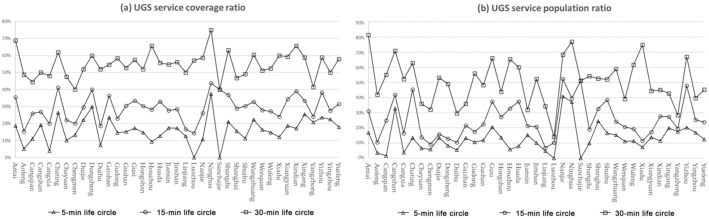
UGS service capability under different traveling scopes. (**a**) UGS service coverage ratio. (**b**) UGS service population ratio.

The effective service area ratio is the ratio of UGS service coverage area to total sub-districts within the study area, which reflects the effective service of UGS in the spatial dimension. Under the three living circle activity ranges, Antai sub-district, Chating sub-district, Dongsheng sub-district and Ninghua sub-district all show a high level, while Aofeng sub-district, Cangxia sub-district, Duihu sub-district and Xiadu sub-district all show a low level. With the increase of activity range, the UGS service coverage of each sub-district was improved, but only Sanchajie sub-district stopped increasing after the travel range reached 1000 m. The results show spatial consistency with accessibility (Figure 8a).

The effective service population ratio is the ratio of the actual number of populations covered by the UGS service range in the spatial unit to the total population, which reflects the actual service of UGS from an objective perspective. None of the three living circle ranges can make the UGS service cover the whole population; with the increase of travel range, the population covered by each sub-district as a whole also tends to increase (Figure 8b).

**Figure 9:**
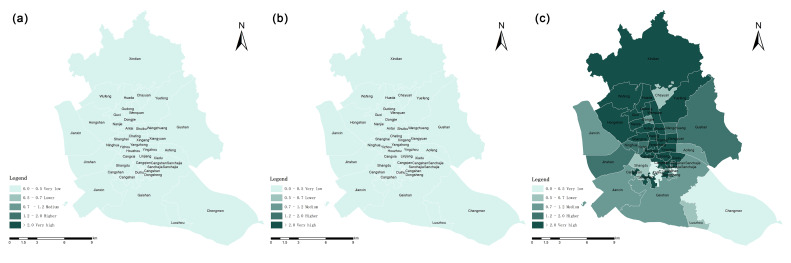
Locational entropy at sub-district scale under different traveling scopes (**a**) 5-min life circle. (**b**) 15-min life circle. (**c**) 30-min life circle.

The results of the sub-district scale locational entropy calculation were matched with the UGS service coverage of each sub-district under different search thresholds to derive the actual equity spatial pattern of UGS distribution. Within the 5-min life circle and the 15-min life circle, all sub-districts in the study area have a locational entropy of less than 1 and are at a low grading level; the per capita acceptance level of UGS is also lower than the overall level (Figure 9).

## 4. Discussion

### 4.1. Similarities and Differences at Different Study Scales

Accessibility at both the residential scale and the sub-district scale showed a spatial distribution pattern of being high in the northwest and low in the southeast. When residential areas are used as the evaluation unit, the residential areas with higher and very high accessibility ratings are mainly distributed in Gulou District and Taijiang District, while the residential areas without accessibility are scattered in all parts of the study area. However, they are mainly concentrated in the underdeveloped areas at the edge of the study area and in some areas with relatively sparse road construction, such as Sanchajie sub-district and Taihu sub-district.

The Gini coefficient shows social inequity at both scales, with inequity more pronounced at the residential scale. This is because the distribution of UGSs is more concentrated in the central part of the study area, where residences have more options and the per capita of UGS is much higher than the average, while some residential areas are not accessible. This creates a serious polarization and an extremely inequitable distribution of UGSs. The evaluation unit of sub-districts is evaluated at the macro level. Each sub-district includes several residential areas, which weakens the characteristics of local area differences.

The results of locational entropy at both evaluation scales indicate that UGS services vary widely among regions in the study area, and there is an obvious spatial mismatch between resources and population. In addition, both scales show an overall spatial distribution pattern of high in the northwest and low in the southeast, but differ in the spatial distribution. When residential areas are used as the evaluation unit, more than 70% of the residential areas receive fewer UGS services than the overall level. At the sub-district scale, more than eight sub-districts are less than the overall level of the study area, accounting for 20.5% of the total number of sub-districts. The difference is that the UGS services of the study area are all at a poor level and are severely under-served at the sub-district scale when the living circle range is small. Additionally, all sub-districts cannot enjoy equitable UGS services within 1000 m. In contrast, some zones can be served by abundant UGS resources at the residential scale. In the sub-district with a low value of locational entropy grading at the macro-scale, there are high value residential units distributed at the micro-scale; it is similar in the sub-district units with good UGS service, containing residential areas with insufficient green space service.

### 4.2. Similarities and Differences at Different Traveling Scopes

Spatial divergence between UGS and population occurs under all three travel scopes. The smaller the activity circle, the more blind areas of UGS services there are. Compared with the 5-min life circle and the 15-min life circle, the values of accessibility result in the 30-min activity circle changing more gently, the number of spatial units with higher accessibility increasing and the service blind areas decreasing. The results show that the spatial accessibility of urban green space will change when the travel range changes. The longer the distance people travel within a certain range, the higher the accessibility; the smaller the variability of green space accessibility between spatial units, the more people will enjoy urban green space services, and the more balanced the distribution of green space resources will be.

In the evaluation of the equity of UGS, all three activity ranges show inequity. As the activity circle range increases, the more the Gini coefficient calculation results converge to 0, the closer the Lorenz curve is to the absolute equity line; that is, the spatial distribution of UGS service supply in Fuzhou is relatively more equal.

With the increase of activity range, the level of UGS service supply in Fuzhou City is significantly improved. When the travel range of residents increases, the effective service area and the population coverage of UGS increases, and the more UGS services residents enjoyed, the better the supply of UGS services.

### 4.3. Application of UGS Planning in Fuzhou

In order to scientifically build an urban green space system and achieve the purpose of equitable supply of services and precise matching of supply and demand, this study mainly proposes the following optimization suggestions in view of the existing problems of UGS allocation in the main urban area of Fuzhou.

(1) The per capita level of UGS resources in Chengmen sub-district, Duihu sub-district and Sanchajie sub-district is much lower than the average level. They are at a disadvantageous level in both scales even under the extreme travel scope. This is mainly due to the incomplete construction, the lack of concentrated distribution of UGS resources, and the inadequate road system. They should be further evaluated and prioritized for measures in urban construction.
(2) For the high population density, the dense distribution of residential areas and land constraints of Chayuan sub-district and Dongjie sub-district, the existing urban layout should make full use of abandoned space to build pocket parks, set up street side green space and corner green space to improve the efficiency of space utilization.
(3) Walking is the primary choice of daily travel for residents. Not only should the construction of UGS areas be strengthened, but also the planning of slow-moving transportation systems should be paid attention to. Due to the sparse road network and inconvenient traffic in Shangdu sub-district, some residents are inconvenienced in using the UGS. The accessibility of UGS for residents should be improved by increasing UGS. Building green corridors connecting with surrounding green space, strengthening road network construction, opening up cut-off roads, and improving road network continuity seem to be enforceable.
(4) In the context of building a livable city in the new era, urban renewal and construction should be tailored to local conditions and strategies should be adopted for different situations. Different regions are not consistent in terms of population structure, economic structure and development status. We should avoid using a single indicator as the basis for spatial optimization when conducting optimization. Urban planners should coordinate and unify with government with considering the city, region and street as a whole at multiple levels. A more scientific and effective construction management system should be formed by satisfying both macroscopic rigid regulations and a microscopic grasp of construction details.
(5) The 5 min, 15 min and 30 min walkable living circles defined in this study determine the spatial evaluation range based on time. They correspond to the basic, neighborhood and daily social and travel distances of residents, respectively. There are also cases of urban spatial planning that emphasize the time-scale as the boundary [28], such as a maximum 5-min walk to all amenities and public transport in Copenhagen [29], the 15-min city in Paris [30], the 20-min neighborhood in the United States [31] and Australia [32], the 20-min town in Singapore [33], and the 15-min convenient living circle in China [28], which have transformed the concepts of urban planning and construction in the past. However, due to the differences in the time delineation of the living circle and the choice of transportation in different countries, a unified spatial scope division has not yet been formed. With the development of intelligent technology, the planning mode of living circle series will undergo new changes. UGS, which has great ecological and social benefits, should bear the brunt; the new planning methods deserve further attention and exploration.

### 4.4. Limitation

Due to the limitations to obtaining data, this study assumed that the population is evenly distributed within the residential area. The number of building floors was not considered. The result calculated from the area weight and the census data is an approximate expression of the number of people in the settlement. In reality, the population distribution is not uniform and should be related to the number of building floors. The accuracy of the research results will be greatly improved if the population data can be accurately obtained by using big data. As a service provider, the higher the quality of the physical environment of the urban green space itself, the more residents can be attracted to visit. Although there have been some studies on the assessment of UGS quality in the past, there is no uniform assessment standard and further discussion should be had. The real meaning of equity should include more consideration of the demand-side state, taking into account the distribution of population, social attributes, and the ability to obtain green space services, etc., which means people-oriented planning. To ensure UGS construction meets the diverse needs and preferences of different social groups is the meaning of UGS equity evaluation.

## 5. Conclusions

This study used web big data, based on G2SFCA, to measure the accessibility of UGS for residents of 39 sub-districts and 2020 residential areas in Fuzhou at different travel scopes, to identify the inequities in the spatial distribution of UGS at the different scales, respectively, and to quantitatively evaluate the actual service of UGS. Finally, scientific proposals were provided for the urban construction and improvement of the urban green space system in Fuzhou. The main conclusions of this study are as follows:

1. Fuzhou city has significant variations in the allocation of urban green space resources with serious social inequities. The accessibility of UGS shows a spatial distribution pattern of being high in the northwest and low in the southeast.
2. Within the walking limit travel distance, with the expansion of residents’ travel range, the accessibility degree of UGS in Fuzhou increases, the distribution of UGS resources is more balanced, therefore the higher the extent of equity, and the level of urban green space service supply is significantly improved.
3. The evaluation results of UGS accessibility, equity, and green space services in the main city of Fuzhou are consistent overall at the residential scale and sub-district scale. However, at smaller travel ranges, the residential scale has an advantage in identifying access to green space resources.
4. The city construction should be planned holistically from multiple levels and take into account the population structure, economic structure and development status, and so on. We should take measures according to local conditions. The construction of UGS should also be further explored and investigated with reference to the planning model of new living circles.

## Figures and Tables

**Table 1:** Classified statistics of UGS.

Type	Count	Ratio/%	Area/ha	Ratio/%
>5 ha	43	50.59	2000.30	96.19
<5 ha	42	49.41	79.24	3.81
Total	85	100.00	2079.54	100.00

**Table 2:** Data display for residential.

Number	District	Sub-District	Population	Number	District	Sub-District	Population
A1	Cangshan	Jinshan	3166	A6	Jinan	Xiangyuan	1254
A2	Jinan	Xindian	663	A7	Taijiang	Shanghai	409
A3	Gulou	Huada	1024	A8	Jinan	Wangzhuang	282
A4	Gulou	Antai	618	A9	Taijiang	Chating	1393
A5	Cangshan	Duihu	1305	A10	Gulou	Gxi	1296

**Table 3:** Traveling scopes.

Type	5-min Life Circle	15-min Life Circle	30-Min Life Circle
Distance	500 m	1000 m	2000 m

**Table 4:** Accessibility grading statistics of residential areas under different traveling scopes.

Level	5-min Life Circle	Ratio	15-min Life Circle	ratio	30-min Life Circle	Ratio
Inaccessible	1689	83.6%	1417	70.1%	907	44.9%
Very low	286	14.2%	496	24.6%	918	45.4%
Lower	27	1.3%	66	3.3%	129	6.4%
Medium	10	0.5%	21	1.0%	56	2.8%
Higher	5	0.2%	17	0.8%	8	0.4%
Very high	3	0.1%	3	0.1%	2	0.1%
Accessible	331	16.4%	603	29.8%	1113	55.1%
Higher than the UGS per capita	190	57.4%	322	53.4%	540	48.5%

**Table 5:** Statistical results of accessibility for different traveling scopes.

	5-min Life Circle	15-min Life Circle	30-min Life Circle
Average	40.2	35.8	32.2
Standard deviation	953.7	337.3	186.0

**Table 6:** Classification statistics of residential location entropy under different traveling scopes.

	Very Low	Lower	Medium	Higher	Very High	Above-Average
Type	0–0.5	0.5–0.75	0.75–1.2	1.2–2.0	>2.0	>1.0
5-min life circle	1802	16	20	30	152	191
15-min life circle	1624	39	55	33	268	323
30-min life circle	1351	75	74	68	451	541

## Data Availability

The data presented in this study are available on request from the author. The data are not publicly available due to privacy. Images employed for the study will be available online for readers.
